# Supplementary material for: Botulinum toxin type A success rates and predictive factors for favourable outcomes in paediatric patients with acquired esotropia
Source: PLoS One. 2025 Sep 12;20(9):e0332471. doi: 10.1371/journal.pone.0332471 (PMC12431342; doi:10.1371/journal.pone.0332471)
Supplement: Attachment — Submitted filename: Response to Reviewers_NR 2025-08-14.docx [file pone.0332471.s002.docx]

**Dear Editor,**

We would like to express our sincere gratitude to you and the reviewers for your thoughtful, constructive, and insightful comments on our manuscript. After carefully considering all of your remarks, we greatly appreciate the kindness, expertise, and genuine concern reflected in your suggestions. Your guidance has been invaluable in enhancing the quality, clarity, and overall impact of our work.

We have carefully addressed each comment and revised the manuscript accordingly. A detailed, point-by-point response to every comment is provided below, including references to the corresponding page and line numbers in the revised manuscript with the track changes file. For ease of review, all changes in the manuscript are clearly highlighted in yellow in the track changes file.We are truly grateful for the opportunity to improve our work and sincerely hope that the updated version meets the high standards of the journal for publication consideration.

**Sincerely,**
Narisa Rattanalert, MD
(Corresponding Author)

**Authors’ responses to Editor’s Comments**

**Editor comments:**

**Comment :** *Emphasize on recurrence, post op diplopia and relapse cases.*

**Response:**

We thank the Editor for this valuable suggestion to further emphasize recurrence, postoperative diplopia, and relapse in our findings. We respectfully note that postoperative diplopia was not assessed in our study; therefore, this outcome could not be analyzed.

In response to your comment, we have clarified and strengthened our reporting of recurrence and relapse, as follows:

**Revised text added to Materials and Methods (Page 5-6, Lines 106-108):**

Recurrent esotropia was defined as the reappearance of esotropia after an initially successful alignment outcome at 6 months following BTXA treatment.

**Revised text added to Results (Page 9, Lines 150-151):**

**Seven patients in the success group (28%) experienced recurrent esotropia following the initial BTXA injection, with a mean time to recurrence of 14.84 ± 3.55 months.**

**Revised text in Discussion (Page 16, Lines 219):**

**Original text:**

Additionally, Yu et al. reported a success rate of 68.68% over a 2-year follow-up for acute acquired comitant esotropia and found that the number of hours spent on near work per day was a significant risk factor for esotropia relapse (Hazard ratio: 1.29, 95% CI = 1.00–1.67) [19].

**Revised text:**

Additionally, Yu et al. reported a success rate of 68.68% over a 2-year follow-up for acute acquired comitant esotropia and found that the number of hours spent on near work per day was a significant risk factor for esotropia recurrence (Hazard ratio: 1.29, 95% CI = 1.00–1.67) [19].

**Authors’ responses to reviewer 1 comments**

**Reviewer #1 Comment 1:**
“Why did you consider 10 PD as a success result? Wouldn’t it be more accurate to cover the fusional range considering the aspect that one of the stability factors after treatment is binocularity recovery? At 10 PD ET diplopia can still be present (half of your cohort had pre-toxin diplopia).”

**Response:**

We sincerely thank the reviewer for this important and insightful observation. The ≤10 PD threshold was selected in accordance with previously published BTXA outcome studies in the strabismus literature, where ocular alignment within this range is widely regarded as clinically satisfactory and generally associated with functional binocular vision in most patients.

We fully agree that recovery of binocularity is an important treatment goal. Accordingly, we assessed binocularity as a secondary outcome. However, due to the retrospective nature of the study, complete binocularity data at 6 months post-BTXA were not available for all patients. Among the 25 patients who achieved a successful motor outcome (≤10 PD) at 6 months, binocularity data were available for 23; of these, 22 demonstrated functional binocular vision, all achieved fusion, and 19 achieved stereopsis, with a median stereopsis of 40 seconds of arc (interquartile range: 25–80).

Given the incomplete binocularity and fusional range data, we could not definitively attribute binocular vision recovery solely to the initial BTXA treatment.

**Revised text added to Results (Page 9, Lines 143-149):**

Among the 25 patients who achieved successful motor outcomes at 6 months, binocularity data were available for 23. Of these, 22 demonstrated functional binocular vision; all achieved fusion, and 19 achieved stereopsis. The median stereopsis was 40 seconds of arc (interquartile range: 25–80).

In the subgroup with acute acquired comitant esotropia, binocularity data were available for 16 of 17 patients. All 16 achieved binocular function, with a median stereopsis of 40 seconds of arc (IQR: 25–80).

**Revised text added to Discussion (Page 17, Lines 252-257):**

Our use of a horizontal deviation within 10 PD as the criterion for treatment success post-BTXA is consistent with previous studies and is generally associated with functional binocular vision. Nevertheless, we acknowledge that diplopia may still occur within this range, and our limited binocularity data at 6 months prevent definitive conclusions. This highlights the importance of incorporating both motor and sensory outcomes when defining treatment success.

**Reviewer #1 Comment 2:**

“I think your data would be more accurate keeping in your statistic only binocular injection.

**Response:**

We sincerely thank the reviewer for this valuable suggestion. In our cohort, binocular BTXA injection was the standard treatment approach, performed in 49 of 51 patients (96.1%).

To address this comment, we excluded the two patients who received unilateral injections and reanalyzed the data including only those who underwent binocular injections.

We have clarified this change in the **Methods** section and incorporated the updated results from the binocular-only analysis into the **Results** section.

**Revised text added to Methods (Page 5, Lines 96-102):**

All patients were administered an intramuscular injection of 7.5 units of BTXA (Dysport®) into the medial rectus muscle in both eyes using forceps, without conjunctival incision or electromyographic guidance, by paediatric ophthalmologists in either the operative room under general anaesthesia or outpatient department under topical anaesthesia. Two patients with esotropia of 30 and 35 PD, who received a unilateral injection of 7.5 units of BTXA into the medial rectus muscle, were excluded to ensure more reliable data and to better assess the effectiveness of BTXA treatment.

**Revised text added to Results (Page 7-14, Lines 127-190):**

Forty-nine patients were included in the study. Table 1, 2, 3 and Figure 1, 2 was updated.

**Table 1.** Baseline characteristics

| **Baseline characteristics** | **N = 49 (%) or mean ± SD** |
| --- | --- |
| Male sex | 32 (65.3) |
| Age at treatment (years) | 8.10 ± 4.02 |
| Symptoms duration before diagnosis (months) | 3.99 ± 5.43 |
| Time of diagnosis to treatment (months) | 4.67 ± 7.27 |
| **Types of acquired esotropia**  Acute acquired comitant esotropia  Intermittent esotropia  Cyclic esotropia  Partially accommodative esotropia  Non-refractive accommodative esotropia | 28 (57.2)  10 (20.4)  6 (12.2)  3 (6.1)  2 (4.1) |
| **Visual acuity (LogMAR)**  Right eye  Left eye | 0.18 ± 0.23  0.15 ± 0.14 |
| **Refraction (dioptres)**  Right eye  Left eye | 0.20 ± 1.89  0.41 ± 1.89 |
| **Amblyopia**  Strabismic amblyopia  Combined strabismic and refractive amblyopia | 8 (16.3)  6  2 |
| **Diplopia**  Yes  No  No available data | 15 (30.6)  20 (40.8)  14 (28.6) |
| Pre-BTXA esotropia at near (PD)  Pre-BTXA esotropia at distance (PD) | 42.55 ± 13.39  42.65 ± 13.35 |
| Follow-up duration (months) | 23.02±16.32 |
| *BTXA* botulinum toxin type A, *LogMAR* logarithm of the minimum angle of resolution, *PD* prism dioptres, *SD* standard deviation | |

**Table 2.** Comparison characteristics between the success and failure groups.

| **Characteristics** | **Success group**  **N=25 (%) or**  **mean ± SD** | **Failure group**  **N=24 (%) or**  **mean ± SD** | ***p-*value*** |
| --- | --- | --- | --- |
| **Sex**  Male  Female | 18 (72.0)  7 (28.0) | 14 (58.3)  10 (41.7) | 0.315 |
| **Age at treatment (years)** | 8.42 ± 4.53 | 7.77 ± 3.48 | 0.578 |
| **Symptom duration (months)** | 4.07 ± 5.47 | 3.92 ± 5.50 | 0.920 |
| **Types of acquired esotropia**  Acute acquired comitant esotropia  Intermittent esotropia  Cyclic esotropia  Partially accommodative esotropia  Non-refractive accommodative esotropia | 17 (70.8)  3 (12.5)  1 (4.2)  2 (8.3)  1 (4.2) | 11 (45.8)  6 (25.0)  5 (20.8)  1 (4.2)  1 (4.2)) | 0.259 |
| **Amblyopia status**  Yes  No | 4 (16.0)  21 (84.0) | 4 (16.7)  20 (83.3) | 1.000 |
| **Pre–BTXA esotropia at near**  ≤ 30 PD  > 30–50 PD  > 50 PD | 40.60 ± 7.12  2 (8.0)  23 (92.0)  0 (0) | 44.58 ± 17.69  8 (33.3)  11 (45.8)  5 (20.8) | 0.303  **0.002** |
| **Pre–BTXA esotropia at distance**  ≤ 30 PD  > 30–50 PD  > 50 PD | 40.4 ± 6.75  3 (12.0)  22 (88.0)  0 (0) | 45.0± 17.69  6 (25.0)  13 (54.2)  5 (20.8) | 0.232  **0.016** |
| **Diplopia**  Yes  No | 10 (50)  10 (50) | 5 (33.3)  10 (66.7) | 0.492 |
| **Time from diagnosis to treatment (months)**  ≥ 2 months  < 2 months | 2.58 ± 3.33  7 (28.0)  18 (72.0) | 6.86 ± 9.45  18 (75.0)  6 (25.0) | **0.038**  **0.001** |
| **Mean follow-up duration (months)** | 15.49 ± 7.86 | 30.86 ± 19.12 | **0.001** |
| **Anaesthesia**  General anaesthesia  Topical anaesthesia | 18 (72.0)  7 (28.0) | 20 (83.3)  4 (16.7) | 0.496 |
| **1-month-post-BTXA alignment**  Within 10 PD  Overcorrection | 10 (50)  10 (50) | 3 (60)  2 (40) | 1.000 |
| *Significant < 0.05 *BTXA* botulinum toxin type A, *PD* prism dioptres, *SD* standard deviation | | | |

**Table 3.** Logistic regression analysis predicting factors of successful outcomes

| **Characteristics** | **Univariate** | | | **Multivariate** | | |
| --- | --- | --- | --- | --- | --- | --- |
|  | OR  (95% CI) | *p* value  (wald-test) | *p* value*  (LR-test) | OR  (95% CI) | *p* value  (wald-test) | *p* value*  (LR-test) |
| **Types of acquired esotropia**  Acute acquired comitant esotropia  Intermittent esotropia  Cyclic esotropia  Partially accommodative esotropia  Non-refractive accommodative esotropia | 1 (reference)  0.43 (0.07, 1.89)  0.13 (0.01, 1.26)  1.29 (0.10, 16.04)  0.65 (0.04, 11.45) | 0.264  0.078  0.841  0.767 | 0.295 |  | ND |  |
| **Amblyopia status**  Yes  No | 1 (reference)  1.05 (0.23, 4.78) | 0.90 | 0.950 |  | ND |  |
| **Pre–BTXA esotropia at near**  ≤ 30 PD  > 30-50 PD | 1 (reference)  8.36 (1.52, 46.15) | 0.015 | **0.007** | 1 (reference)  6.97 (1.10, 44.13) | 0.039 | **0.008** |
| **Pre–BTXA esotropia at distance**  ≤ 30 PD   > 30-50 PD | 1 (reference)  3.38 (0.72, 15.89) | 0.122 | 0.111 |  | NS |  |
| **Diplopia**  Yes  No | 1 (reference)  0.50 (0.13, 1.99) | 0.327 | 0.322 |  | ND |  |
| **Time from diagnosis to treatment**  ≥ 2 months  < 2 months | 1 (reference)  7.71 (2.16, 27.50) | 0.002 | **0.001** | 1 (reference)  6.31 (1.51, 26.41) | 0.012 | **0.001** |
| **Time from symptom to treatment**  ≥ 3 months  < 3 months | 1 (reference)  2.81 (0.73, 10.84) | 0.133 | 0.122 |  | NS |  |
| **1-month-post-BTXA alignment**  Within 10 PD  Overcorrection | 1 (reference)  1.50 (0.20, 10.99) | 0.690 | 0.688 |  | ND |  |
| *Significant < 0.05  *CI* confidence interval, *OR* odd ratio, *ND* not done, *NS* not significant, *BTXA* botulinum toxin type A, *PD* prism dioptres | | | | | | |

**Fig. 1** Kaplan–Meier analysis estimated the probability of treatment success for 49 patients as 51%, 48%, 28%, and 21% at 6-, 12-, 18-, and 24-months intervals, respectively.

**Fig. 2** Kaplan–Meier analysis estimated the probability of treatment success categorised by the range of pre-BTXA esodeviation at near (a) and distance (b), as well as time from diagnosis to treatment (c).

**Reviewer #1 Comment 3:**

In my opinion subsequent interventions are not relevant considering the study purpose.”

**Response:**

We sincerely thank the reviewer for this thoughtful comment. We agree that the primary evaluation should focus on the initial BTXA injection to accurately reflect its standalone efficacy. In our original analysis, subsequent interventions were included only to provide a complete clinical overview of patient management.

In response to your suggestion, we have:

- Clarified in the **Methods** that the primary outcome analysis was restricted to the results of the initial BTXA treatment.
- Presented the outcomes of secondary interventions separately in the **Results** section, ensuring they do not influence or confound the primary success rate calculation.

**Revised text added to Methods (Page 6, Lines 118-120):**

Primary outcome analysis was restricted to the results following the initial BTXA injection. Data from subsequent interventions were presented separately for descriptive purposes and were not included in the primary success rate calculation.

**Revised text added to Results (Page 9, Lines 142-143):**

The success rate of initial BTXA treatment at 6 months was 51% (25/49) in the overall cohort and 60.7% (17/28) among patients with acute acquired comitant esotropia.

**Reviewer #1 Comment 4:**
“It is possible that stability after treatment is influenced by other factors such as lifestyle. Nowadays we confront Acute Esotropia related to screen overuse, especially smartphones; recurrence of ET is not unusual after BT or surgery, so some other factors can be involved in the success rate at two years after injection.”

**Response:**

We sincerely thank the reviewer for this insightful observation. We agree that lifestyle factors—particularly excessive near work and prolonged screen use—may influence both the stability of alignment and the risk of recurrence following BTXA treatment. Although our retrospective dataset did not include quantified measurements of lifestyle habits, we recognize the importance of these factors and have addressed them in the revised **Discussion** section, supported by relevant literature.

**Revised text added to Discussion (Page 17, Lines 257-263):**

In addition to the identified predictive factors, lifestyle-related visual demands, such as prolonged near work and smartphone use, may influence both initial treatment outcomes and long-term stability. Previous reports have linked excessive screen use with acute acquired comitant esotropia and higher recurrence rates following BTXA or surgery. Although such data were not available in our cohort, these factors warrant further investigation. Therefore, future prospective, multicenter randomized controlled trials with standardized follow-up protocols are recommended to establish more robust evidence supporting the efficacy and safety of BTXA in childhood-acquired comitant esotropia.

**Authors’ responses to reviewer 2 comments**

**Reviewer #2 Comment 1:**
“Line 133–35: It would have been better to mention the recurrence rate? 7 patients had recurrence.”

**Response:**

We sincerely thank the reviewer for this helpful suggestion. In response, we have explicitly reported the recurrence rate, including the proportion of affected patients and the mean time to recurrence, in the **Results** section.

**Revised text added to Results (Page 9, Lines 150-151):**

Seven patients in the success group (28%) experienced recurrent esotropia following the initial BTXA injection, with a mean time to recurrence of 14.84 ± 3.55 months.

**Reviewer #2 Comment 2:**
“Current study brings out the fact that there is 51% success rate with BTXA treatment, with a trend of a decline in success over time. Interestingly only one-fourth of the patients sustained optimal outcomes beyond 2 years.”

**Response:**

We sincerely thank the reviewer for this accurate and important observation. We agree that our findings demonstrate a 51% success rate at 6 months, with a progressive decline in success over time, and that only a minority of patients maintained optimal alignment beyond two years. To emphasize this point, we have added a statement to the **Discussion** highlighting the long-term outcome trends and their clinical implications.

**Revised text added to Discussion (Page 15, Lines 207-212):**

In this regard, our study revealed a 51% success rate with BTXA treatment, showing a decline in success over time. Nevertheless, approximately one-fifth of the patients sustained optimal outcomes beyond 2 years. Our Kaplan–Meier analysis confirmed a progressive decline in the probability of treatment success over time, with only 21% of patients maintaining optimal ocular alignment beyond two years. This trend underscores the importance of long-term follow-up to detect and manage recurrence in a timely manner.

**Reviewer #2 Comment 3:**
“This study does not come up with new knowledge apart from most effective for small-to-moderate angle deviations which is very much known looking at the current literature.”

**Response:**

We sincerely thank the reviewer for this observation. We acknowledge that our finding regarding the association between smaller-to-moderate pre-BTXA deviation angles and better outcomes is consistent with existing literature. However, our study offers additional contributions by:

- Identifying a narrower predictive deviation range (>30–50 PD) associated with optimal BTXA success.
- Quantifying the effect of early intervention (<2 months from diagnosis) as an independent predictor of success in a multivariate logistic regression model.

We have clarified these novel aspects in the **Discussion** to better highlight our study’s contribution to the field.

**Revised text added to Discussion (Page 18, Lines 266-270):**

While our observation that smaller-to-moderate angle deviations respond more favorably to BTXA is consistent with prior reports, our multivariate analysis further refined the predictive range to >30–50 PD for optimal outcomes. Additionally, early intervention within 2 months of diagnosis emerged as an independent predictor of success, reinforcing the value of prompt referral and treatment.

**Reviewer #2 Comment 4:**
“Overall, the analysis of factors using univariate model underscores the potential benefits of BTXA as an alternative treatment for acquired esotropia, and of course this possibly due to the retrospective nature of the study and overall small sample size representing each group of esotropia and less duration of follow up.”

**Response:**

We sincerely thank the reviewer for this constructive comment. We agree that the retrospective, single-arm design, relatively small subgroup sizes, and variable follow-up durations are important limitations of our study. To address this, we have explicitly acknowledged these constraints in the **Discussion** and have outlined recommendations for future research to strengthen the evidence base.

**Revised text added to Limitations section of Discussion (Page 17-18, Lines 250-252, 262-264):**

However, a notable limitation of this study was its retrospective, single-arm design, relatively small subgroup sizes for certain esotropia types, and variable follow-up durations.

Therefore, future prospective, multicenter randomized controlled trials with standardized follow-up protocols are recommended to establish more robust evidence supporting the efficacy and safety of BTXA in childhood-acquired comitant esotropia.
